# Prognostic nutritional index value in the prognosis of Kawasaki disease with coronary artery lesions

**DOI:** 10.3389/fnut.2023.1075619

**Published:** 2023-02-01

**Authors:** Jie Liu, Danyan Su, Piaoliu Yuan, Yuqin Huang, Bingbing Ye, Kaizhi Liang, Yusheng Pang

**Affiliations:** Department of Pediatrics, First Affiliated Hospital, Guangxi Medical University, Nanning, China

**Keywords:** Kawasaki disease, coronary artery lesion, prognostic nutritional index, nutritional status, prognosis

## Abstract

**Objectives:**

The prognostic nutritional index (PNI) is a purported predictor of intravenous immunoglobulin (IVIG) resistance and coronary artery aneurysm (CAA) development in patients with Kawasaki disease (KD). However, limited data exist on CAA regression. This study aimed to confirm whether the PNI is a predictor for CAA persistency in patients with KD.

**Methods:**

This retrospective study grouped 341 patients with KD based on the coronary artery status and time of aneurysm persistence. The clinical and laboratory parameters were compared, and multivariate logistic regression analysis was performed to identify the independent risk factors for persistent CAA. The receiver operating characteristic (ROC) curve was further used to assess the predictive values of the PNI in persistent CAA.

**Results:**

Among the study patients, 80 (23.5%) presented with CAA, including CAA persisting for 2 years in 17 patients (5.0%). Patients with CAA were more frequently treated with corticosteroids (*p* < 0.016). No statistically significant differences were found in the nutritional status and PNI among patients with or without coronary artery lesions, regardless of injury severity. Patients in the persistent CAA group presented with higher rates of overnutrition and showed lower PNI values and a higher incidence of thrombosis than those in the normal group (*p* < 0.05). The PNI and the maximum *Z*-score at 1 month of onset were significantly associated with CAA persisting for 2 years and may be used as predictors of persistent CAA. The area under the ROC curve was 0.708 (95% confidence interval, 0.569–0.847), and a 40.2 PNI cutoff yielded a sensitivity and specificity of 41 and 92%, respectively, for predicting CAA persisting for 2 years. Kaplan–Meier survival analysis revealed that the estimated median time of aneurysm persistence was significantly higher in patients with PNI values of ≤40 than in those with PNI values of >40 (hazard ratio, 2.958; 95% confidence interval, 1.601–5.464; *p =* 0.007). After sampling-time stratification, the PNI differed significantly between patients with and without persistent CAA when sampled on the second (*p* = 0.040), third (*p* = 0.028), and fourth days (*p* = 0.041) following disease onset.

**Conclusion:**

A lower PNI value is an independent risk factor for CAA persisting for 2 years in patients with KD, besides the maximum *Z*-score at 1 month after onset. Furthermore, the PNI obtained within 4 days from fever onset may possess greater predictive power for patients with persistent CAA.

## 1. Introduction

Kawasaki disease (KD) is an acute self-limiting systemic vascular disease that causes serious cardiac complications, such as coronary artery lesions (CALs), in young children. The administration of intravenous immunoglobulin (IVIG) together with aspirin has greatly reduced the risk of CALs (1). However, 20–30% of patients with KD develop transient coronary artery dilatation (CAD), and 5–9% will develop coronary artery aneurysms (CAA) despite timely treatment (2, 3). The duration of CAA is strongly associated with long-term prognosis (4–6), and approximately 50% of aneurysmal coronary segments regress within the first or second year (5–9). Long-term management and poor outcomes in patients with KD and CAA are significant. Advani et al. (10) reported that 77.4% of KD-related aneurysms could return to normal within 2 years of onset; however, while CAA persists, thrombosis, CAA rupture, myocardial infarction, and heart failure may occur, inevitably affecting the children’s quality of life and endangering their lives. Therefore, the timing of the regression of coronary vasculitis is crucial for future prognosis stratification methods.

Multisystem inflammatory syndrome in children, which has similar etiopathogenesis and overlapping clinical features as KD, may also contribute to CAA, especially in obese patients (11–13). Obesity increases the risk of incidence of CALs by affecting the metabolism and acting as a risk factor for coronary artery disease (14–16). Overnutrition, namely overweight or obesity, is a pro-inflammatory state, whereas systemic inflammation and immune responses play a crucial role in the pathogenesis and progression of KD. Recently, the prognostic nutritional index (PNI), based on a combination of albumin levels and peripheral blood lymphocyte count, has been associated with the nutritional, immunological, and inflammatory statuses of the body (17, 18). Several studies have associated a low pre-treatment PNI with a high incidence of CALs and IVIG resistance in patients with KD (19–23); however, the association between nutritional status and PNI in relation to CAA regression remains elusive. Therefore, the present study was designed to determine whether the PNI could predict persistent CAA in patients with KD.

## 2. Materials and methods

### 2.1. Study design and population

In total, 341 children with KD who received IVIG treatment within 10 days from fever onset were admitted to the Pediatrics Department of the First Affiliated Hospital of Guangxi Medical University, China, between 2013 and 2020. The diagnostic criteria for KD were based on the adapted American Heart Association (AHA) guidelines (1, 24) as revised by the Japanese diagnostic guidelines, 6th revision (25). A pediatric cardiologist and experienced pediatric echocardiographers determined the diagnosis and measured CAAs, respectively. CALs were defined according to *Z*-scores adjusted for the body surface area of a coronary artery with an internal diameter of ≥2.0 in the proximal right coronary artery, left main coronary artery, or left anterior descending artery. Only cases in which the maximal *Z*-score persisted at ≥2.5 for longer than a month after disease onset were considered CAA cases, and those with a maximal *Z*-score between 2.5 and 2.0 were considered CAD cases, as described in the AHA guidelines (1). For patients with echocardiography follow-up, CAA regression was defined as the normal appearance and size (*Z*-score of <2.5) of all coronary arteries as shown by echocardiography images, in addition to normal cardiac function (1). Nutritional status was classified as malnutrition (<median − 2SD), normal (median − 2SD – median + 1SD), and overnutrition (>median + 1SD) based on the Chinese growth standard (26). Patients were excluded if they had recurrent KD, as the previous CAA could have persisted as a coronary complication. Furthermore, patients who did not receive IVIG treatment or received IVIG treatment after 10 days from fever onset, patients who received initial IVIG or corticosteroid (oral, intravenous, intramuscular, or subcutaneous) therapy at another hospital with incomplete or occasionally unavailable data required for statistical analyses, and patients with incomplete outcomes or sonographic data were also excluded.

All patients were treated according to the AHA guidelines (1, 24). IVIG resistance was considered when patients showed symptoms of persistent or recrudescent fever (axillary or rectal temperatures of ≥37.5°C and ≥ 38.0°C, respectively) for ≥36 h, but ≤7 days after receiving the initial IVIG infusion (2 g/kg) (1), and received a second dose of gamma globulin therapy. Owing to unremitting fever after the completion of the second IVIG treatment, methylprednisolone was administered by intravenous injection at a dose of 2 mg/kg, twice per day. Subsequently, the administration was tapered and withdrawn until there was normalization of C-reactive protein levels. Oral prednisolone administration was initiated at 2 mg/kg, decreased to 1 mg/kg, and finally reduced to 0.5 mg/kg per day and tapered for ≥2 weeks. All patients were first classified into the following three groups: no coronary artery lesion (NCAL; *n* = 145), CAD (*n* = 116), and CAA (*n* = 80). Among the 80 patients with CAA and echocardiography follow-up data, 63 patients experienced CAA regression within 2 years of acute illness and were assigned to the normal group. The remaining 17 patients with CAA persisting for 2 years were defined as the persistent CAA group.

This research was approved by the medical ethics committee of the First Affiliated Hospital of Guangxi Medical University (code number: 2021[KY-E-240]). Written informed consent was waived as this was a retrospective study.

### 2.2. Data collection

Data regarding the demographic, clinical, and laboratory characteristics were documented as follows: (1) general demographic data, including age (months) at disease onset, sex, body mass index (BMI), and nutritional status; (2) clinical characteristics, including the prevalence of incomplete KD, duration of fever before admission, illness day at treatment (considering the first day of fever as day 1 of illness), and response to IVIG therapy; and (3) laboratory indicators, such as white blood cell count, neutrophil count, lymphocyte count, hemoglobin concentration, platelet count, aspartate aminotransferase level, alanine aminotransferase level, total bilirubin level, serum albumin and sodium concentrations, and C-reactive protein concentration. All laboratory indicators were collected for assessment during the acute febrile period and prior to initial IVIG treatment. The neutrophil-to-lymphocyte count ratio (NLR), platelet-to-lymphocyte count ratio, albumin-to-globulin (A/G) ratio, and C-reactive protein-to-albumin ratio were calculated. The PNI was calculated using serum albumin concentration and lymphocyte count [PNI = albumin (g/L) + 5 × lymphocyte count (×10^9^/L)]. The systemic immune-inflammation index (SII) was calculated using the neutrophil, lymphocyte, and platelet counts [SII = platelet count × (neutrophil count/lymphocyte count)]. Echocardiography was routinely performed during the acute phase before IVIG treatment and was repeated at 1, 3, 6, 12, and 24 months after the onset of fever or until CAAs had returned to their normal size. *Z*-scores were calculated using Dallaire equations (27) and plotted against time to assess the evolution of CAA size over time.

### 2.3. Statistical analysis

The data distribution normality was verified using the Shapiro–Wilk and homogeneity tests. Normally distributed data were expressed as means with standard deviations. Two-independent-sample *t*-tests or one-way analysis of variance were performed to compare the data between the groups. Measurements of continuous data without normal distribution were expressed as medians (four-digit interval) [*P_50_* (*P_25_, P_75_*)] and compared between groups using the Mann–Whitney *U* or Kruskal–Wallis *H* tests. Categorical data were expressed as percentages (%). Fisher’s exact, chi-squared, or Pearson’s chi-squared tests were used to perform intergroup comparisons, and the Bonferroni correction was applied for multiple comparisons. Variance inflation factors were used for checking collinearity and significant indices were analyzed using multivariate logistic regression analysis to determine risk factors. The optimum threshold for the significant parameter was constructed using receiver operating characteristic (ROC) curves. The Kaplan–Meier survival analysis was performed to compare the recovery time between the cohorts using the log-rank test, and two-tailed *p*-values were calculated, with a *p*-value of <0.05 considered significant. All statistical analyses were performed using IBM SPSS for Windows, version 26 (IBM Corp., Armonk, NY, United States).

## 3. Results

### 3.1. Baseline characteristics

A total of 341 children who were hospitalized for KD during the study period were analyzed. The participants’ ages ranged from 2 to 117 months, and 56 (16.4%) children had primary IVIG resistance. Among them, 21 (6.4%) children received corticosteroid therapy in addition to the second dose of gamma globulin therapy. Almost all children in our cohort were treated with aspirin, and none received additional treatment such as infliximab, cyclosporine, anakinra, cyclophosphamide, or plasma exchange during this period. Although 80 (23.5%) children developed CAA, no major adverse cardiovascular events were observed in any patient over a median follow-up period of 2 years.

### 3.2. Comparison of baseline characteristics between the KD groups based on the *Z*-scores

The NCAL group had a higher prevalence of incomplete KD and a lower proportion of male patients than the other groups. Corticosteroid treatment was more common in the CAA group than that in the other groups. Moreover, the CAA group had more patients aged <1 year than the NCAL group did (*p* < 0.016; Table 1). However, statistically significant differences were not observed between the groups in terms of BMI, nutritional status, duration of fever before admission, illness day at treatment, IVIG resistance, and laboratory indicators (all *p* > 0.05).

**Table 1:** Demographic and clinical characteristics of the KD patients per CAA group based on *Z*-scores.

	NCAL (*n* = 145)	CAD (*n* = 116)	CAA (*n* = 80)
Demographic characteristics			
Age (months)	27.00 (16.00, 48.50)	23.00 (13.25, 34.00)	21.00 (11.25, 36.00)
<12 months	16 (11.0)	18 (15.5)	20 (25.0)^*^
Male	87 (60.0)	88 (75.9)^*^	65 (81.3)^*^
BMI (kg/m^2^)	15.51 ± 1.69	15.47 ± 1.54	15.70 ± 1.65
Nutritional status			
Normal	114 (78.6)	90 (77.6)	63 (78.8)
Malnutrition	13 (9.0)	16 (13.8)	8 (10.0)
Overnutrition	18 (12.4)	10 (8.6)	9 (11.3)
Clinical characteristics			
Incomplete KD	49 (33.8)	12 (10.3)^*^	10 (12.5)^*^
Fever duration before admission (day)	5.00 (4.00, 7.00)	6.00 (5.00, 7.00)	6.00 (4.00, 7.00)
Days of illness at primary treatment (day)	6.00 (5.00, 8.00)	7.00 (6.00, 8.00)	7.00 (6.00, 8.00)
IVIG resistance	22 (15.2)	16 (13.8)	18 (22.5)
Corticosteroid therapy	6 (4.1)	4 (3.4)	11 (13.8)^*^^,^^†^
Laboratory parameters			
White blood cell count (×10^9^/L, ref. 5–12 × 10^9^/L)	13.58 (9.96, 17.09)	13.97 (9.55, 20.65)	14.41 (10.35, 19.63)
Neutrophils count (×10^9^/L, ref. 1.8–6.3 × 10^9^/L)	8.72 (5.48, 12.50)	8.80 (5.29, 13.42)	8.53 (4.90, 13.35)
NLR	2.70 (1.66, 5.53)	2.49 (1.36, 3.96)	2.78 (1.14, 4.96)
Hemoglobin (g/L, ref. 120–160 g/L)	107.49 ± 12.18	106.12 ± 13.61	107.57 ± 15.89
≤110 g/L	85 (58.6)	71 (61.2)	43 (53.8)
Platelet count (×10^12^/L, ref. 125–350 × 10^12^/L)	353.55 ± 136.78	346.88 ± 141.57	338.48 ± 158.27
PLR	112.22 (80.13, 176.85)	98.88 (69.07, 142.86)	100.00 (52.35, 142.86)
CRP (mg/L, ref. 0–10 mg/L)	69.92 (28.97, 121.99)	57.81 (20.43, 99.08)	79.78 (34.22, 130.53)
Sodium (mmol/L, ref. 137–147 mmol/L)	135.94 ± 3.09	136.55 ± 3.12	135.84 ± 3.19
ALT (U/L, ref. 7–45 U/L)	28.00 (17.00, 63.00)	28.00 (16.00, 68.75)	34.00 (20.00, 80.50)
AST (U/L, ref. 13–40 U/L)	34.00 (24.00, 45.00)	32.00 (25.25, 45.00)	34.50 (26.25, 53.75)
Total bilirubin (μmol/L, ref. 3.4–20.5 μmol/L)	5.00 (3.10, 8.85)	6.72 (3.93, 9.40)	6.30 (4.13, 10.60)
Albumin (g/L, ref. 40–55 g/L)	35.82 ± 5.05	34.84 ± 5.99	34.01 ± 5.86
<35 g/L	65 (44.8)	57 (49.1)	48 (60.0)
A/G ratio	1.35 ± 0.47	1.34 ± 0.51	1.29 ± 0.56
CRP/albumin ratio	1.95 (0.79, 3.70)	1.69 (0.60, 3.15)	2.30 (0.94, 4.51)
PNI	52.30 ± 11.91	54.56 ± 13.99	53.15 ± 13.26
SII	980.99 (531.91, 1906.63)	924.59 (396.60, 1378.43)	873.31 (319.22, 1730.59)

Data are expressed as means with standard deviations, medians (IQR), or as frequency (percentage). *p*-Value < 0.016 was considered statistically significant after Bonferroni correction for multiple comparisons. CAA, coronary artery aneurysm; BMI, body mass index; KD, Kawasaki disease; IVIG, intravenous immunoglobulin; NLR, neutrophil-to-lymphocyte count ratio; PLR, platelet-to-lymphocyte count ratio; CRP, C-reactive protein; ALT, alanine aminotransferase; AST, aspartate aminotransferase; A/G, albumin-to-globin; PNI, prognostic nutritional index; SII, systemic immune-inflammation index.

* Statistically significant versus NCAL group.

† Statistically significant versus CAD group.

### 3.3. Comparison between the CAA groups based on the risk factors for persistent CAA

The neutrophil count, NLR, and maximum *Z*-score at baseline and 1 month after onset were significantly higher in the persistent CAA group, whereas the serum albumin concentration, A/G ratio, and PNI were lower than those in the normal group (*p* < 0.05). Regarding the nutritional status and clinical outcomes, the persistent CAA group had more patients with abnormal nutritional status and showed a higher incidence of thrombosis than the normal group, with statistically significant differences (*p* < 0.05), as shown in Table 2. The multivariate logistic regression analysis included nutritional status, A/G ratio, PNI, and maximum *Z*-score at baseline and 1 month after onset plus the SII, which was previously reported as a risk factor for coronary artery disease and cardiovascular complications (21, 28–30). The neutrophil count, NLR, and serum albumin concentration had collinearity with PNI and SII; thus, they were excluded. No collinearity was present among the analyzed factors, and the PNI [odds ratio (OR), 0.909; 95% confidence interval (CI): 0.836–0.988] and the maximum *Z*-score at 1 month of onset (OR, 4.292; 95% CI: 1.971–9.350) were identified as independent risk factors for CAA persisting for 2 years. In model 1, we only adjusted for age and sex and found that compared with the normal group, the PNI [adjusted odds ratio (aOR), 0.897; 95% CI: 0.817–0.985] and maximum *Z*-score at 1 month of onset (aOR, 5.037; 95% CI: 2.035–12.467) were associated with increased odds of persistent CAA. In model 2, we also adjusted for IVIG resistance and corticosteroid therapy, and found that an additional adjustment for laboratory indices changed the observed association only minimally; the aORs for PNI and maximum *Z*-score at 1 month were 0.891 and 5.631, respectively (Table 3).

**Table 2:** Demographic and clinical characteristics of the patients with CAA between groups based on the prognosis.

	Total (*n* = 80)	Persistent CAA group (*n* = 17)	Normal group (*n* = 63)	*p*-Value
Demographic characteristics				
Age (months)	21.00 (11.25, 36.00)	36.00 (11.00, 80.50)	20.00 (11.00, 33.00)	0.102
<12 months	20 (25.0)	4 (23.5)	16 (25.4)	1.000
Male	65 (81.3)	15 (88.2)	50 (79.4)	0.630
BMI (kg/m^2^)	15.52 (14.53, 16.44)	15.62 (14.63, 17.76)	15.43 (14.53, 16.12)	0.335
Nutritional status				0.045
Normal	63 (78.8)	10 (58.8)	53 (84.1)	
Malnutrition	9 (11.3)	3 (17.6)	6 (9.5)	
Overnutrition	8 (10.0)	4 (23.5)	4 (6.3)	
Clinical characteristics				
Incomplete KD	12 (15.0)	2 (11.8)	10 (15.9)	0.969
Fever duration before admission (day)	6.00 (4.00, 7.00)	7.00 (3.00, 7.50)	5 (4.00, 7.00)	0.565
Days of illness at primary treatment (day)	6.90 ± 2.07	7.24 ± 1.92	6.81 ± 2.11	0.454
IVIG resistance	18 (22.5)	7 (41.2)	11 (17.5)	0.080
Corticosteroid therapy	11 (13.8)	5 (29.4)	6 (9.5)	0.086
Laboratory parameters				
White blood cell count (×10^9^/L, ref. 5–12 × 10^9^/L)	15.06 ± 6.82	17.56 ± 7.90	14.38 ± 6.40	0.089
Neutrophils count (×10^9^/L, ref. 1.8–6.3 × 10^9^/L)	9.68 ± 6.15	12.97 ± 6.71	8.79 ± 5.73	0.012
NLR	2.78 (1.14, 4.96)	4.23 (2.77, 9.20)	2.38 (0.80, 4.45)	0.009
Hemoglobin (g/L, ref. 120–160 g/L)	107.57 ± 15.89	109.71 ± 14.68	107.00 ± 16.27	0.535
≤110 g/L	43 (53.8)	8 (47.1)	35 (55.6)	0.533
Platelet count (×10^12^/L, ref. 125–350 × 10^12^/L)	338.48 ± 158.27	337.54 ± 138.84	338.73 ± 164.14	0.978
PLR	100.00 (52.35, 142.86)	134.59 (80.67, 182.18)	97.88 (48.93, 142.86)	0.425
CRP (mg/L, ref. 0–10 mg/L)	79.78 (34.22, 130.53)	112.69 (54.72, 126.35)	73.10 (27.62, 137.40)	0.438
Sodium (mmol/L, ref. 137–147 mmol/L)	135.84 ± 3.19	135.19 ± 3.57	136.02 ± 3.06	0.337
ALT (U/L, ref. 7–45 U/L)	34.00 (20.00, 80.50)	53.00 (32.00, 94.00)	26.00 (19.00, 65.00)	0.052
AST (U/L, ref. 13–40 U/L)	34.50 (26.25, 53.75)	31.50 (26.00, 79.50)	35.00 (27.00, 53.00)	0.855
Total bilirubin (μmol/L, ref. 3.4–20.5 μmol/L)	6.30 (4.13, 10.60)	7.80 (4.40, 18.15)	6.30 (4.10, 10.00)	0.300
Albumin (g/L, ref. 40–55 g/L)	34.01 ± 5.86	30.76 ± 5.81	34.89 ± 5.61	0.009
<35 g/L	48 (60.0)	15 (88.2)	33 (52.4)	0.007
A/G ratio	1.29 ± 0.56	1.05 ± 0.52	1.36 ± 0.55	0.042
CRP/albumin ratio	2.30 (0.94, 4.51)	3.23 (1.64, 4.45)	2.17 (0.86, 4.64)	0.199
PNI	53.15 ± 13.26	45.63 ± 11.02	55.18 ± 13.15	0.008
SII	873.31 (319.22, 1730.59)	1647.67 (645.55, 2403.74)	770.00 (259.20, 1287.49)	0.087
Maximum *Z*-score				
Baseline	3.87 (3.14, 4.61)	4.63 (4.02, 5.50)	3.54 (3.06, 4.13)	0.002
1 month later	3.95 ± 1.46	5.71 ± 1.61	3.48 ± 0.98	< 0.001
Outcomes				
Thrombosis	12 (15.0)	8 (47.1)	4 (6.3)	< 0.001

Data are expressed as means with standard deviations, medians (IQR), or as frequency (percentage). CAA, coronary artery aneurysm; BMI, body mass index; KD, Kawasaki disease; IVIG, intravenous immunoglobulin; NLR, neutrophil-to-lymphocyte count ratio; PLR, platelet-to-lymphocyte count ratio; CRP, C-reactive protein; ALT, alanine aminotransferase; AST, aspartate aminotransferase; A/G, albumin-to-globin; PNI, prognostic nutritional index; SII, systemic immune-inflammation index.

**Table 3:** Relationship between risk factors and the persistent CAA.

Characteristic	Univariable		Multivariable			Adjusted odds ratios^a^ (95%CI)			
	Odds ratios (95% CI)	*p-*Value	Odds ratios (95% CI)	*p-*Value	VIF	Model 1	*p-*Value	Model 2	*p-*Value
Nutritional status (3 groups)					1.050				
Normal	1 (reference)		1 (reference)			1 (reference)		1 (reference)	
Malnutrition	2.650 (0.567–12.384)	0.215	6.118 (0.447–83.819)	0.175		11.231 (0.680–185.534)	0.091	10.029 (0.575–175.020)	0.114
Overnutrition	5.300 (1.134–24.769)	0.034	6.876 (0.740–63.919)	0.090		4.112 (0.280–60.480)	0.303	2.893 (0.169–49.399)	0.463
A/G ratio	0.307 (0.096–0.984)	0.047	0.599 (0.076–4.732)	0.627	1.213	0.648 (0.061–6.881)	0.719	0.496 (0.040–6.162)	0.585
PNI	0.932 (0.881–0.985)	0.012	0.909 (0.836–0.988)	0.024	1.340	0.897 (0.817–0.985)	0.022	0.891 (0.799–0.993)	0.037
SII	1.000 (1.000–1.001)	0.099	1.000 (1.000–1.001)	0.453	1.253	1.000 (1.000–1.001)	0.528	1.000 (0.999–1.001)	0.460
Maximum *Z*-score (baseline)	2.262 (1.277–4.006)	0.005	1.304 (0.637–2.669)	0.467	1.615	1.408 (0.667–2.970)	0.369	1.284 (0.572–2.883)	0.545
Maximum *Z*-score (1 month later)	4.153 (2.169–7.950)	<0.001	4.292 (1.971–9.350)	<0.001	1.684	5.037 (2.035–12.467)	<0.001	5.631 (2.013–15.753)	0.001

CAA, coronary artery aneurysm; CI, confidence interval; VIF, variance inflation factors; A/G, albumin-to-globin; PNI, prognostic nutritional index; SII, systemic immune-inflammation index.

a Model 1: adjusted for age (months) and sex; Model 2: model 1 plus adjusted for intravenous immunoglobulin resistance and corticosteroid therapy.

### 3.4. Predictive value for CAA with a poor prognosis

**Figure 1:**
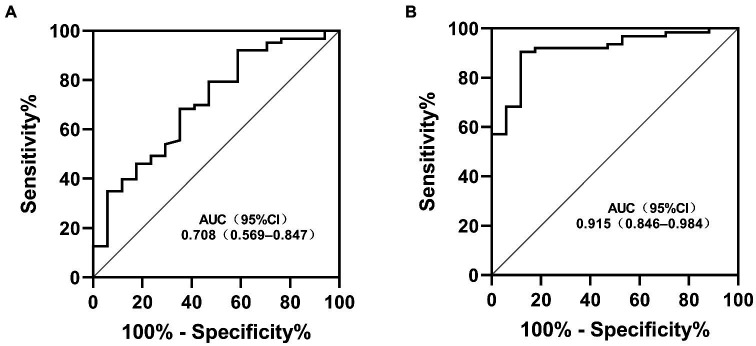
Receiver operating characteristic curves for the risk factors in patients with KD and persistent CAA **(A)** Prognostic nutritional index; **(B)** Maximum *Z*-score at 1 month. KD, Kawasaki disease; CAA, coronary artery aneurism; AUC, area under the ROC curve; CI, confidence interval.

**Figure 2:**
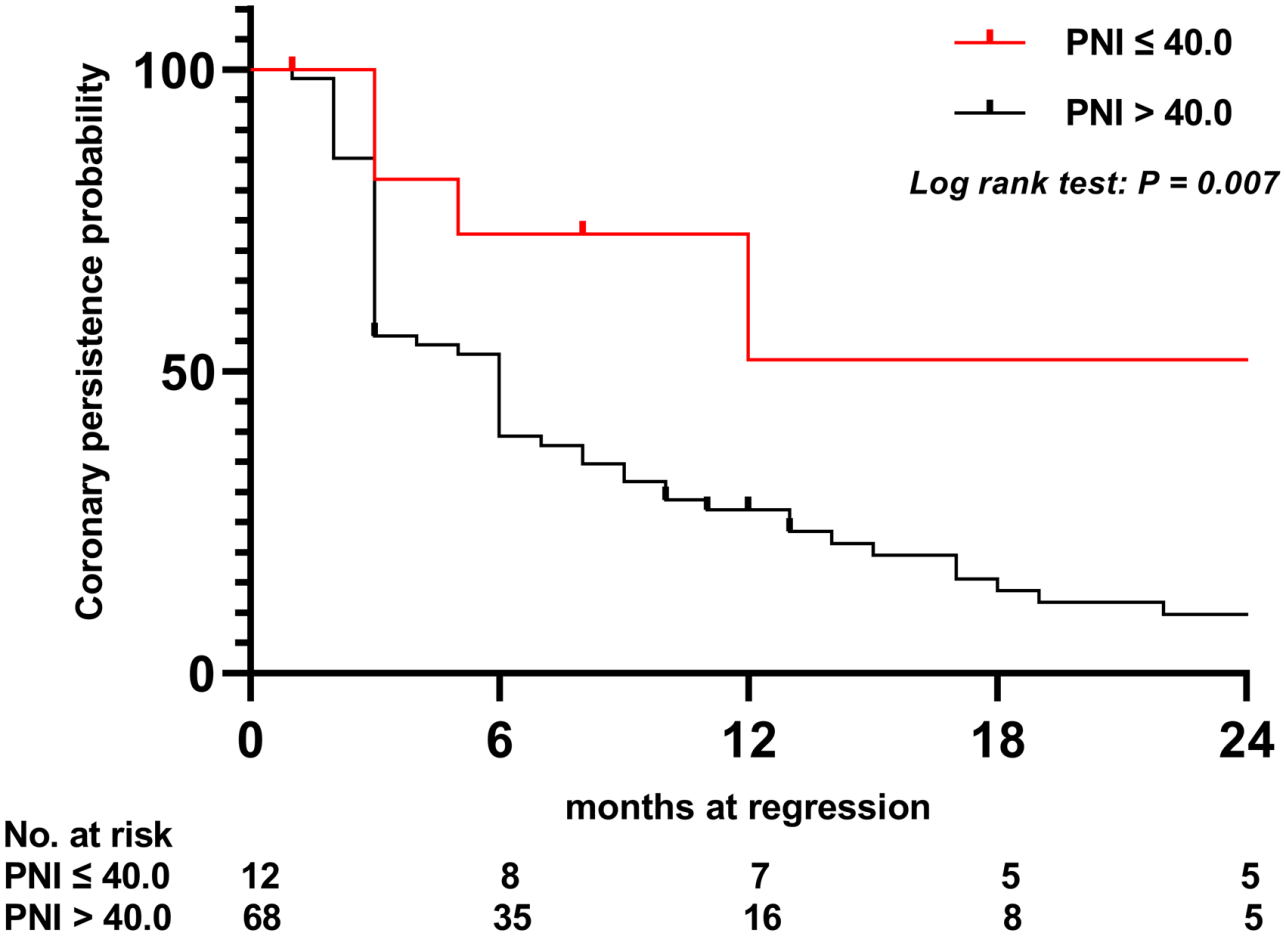
Kaplan–Meier comparative survival analysis of the time to coronary artery dilatation normalization. PNI, prognostic nutritional index.

**Figure 3:**
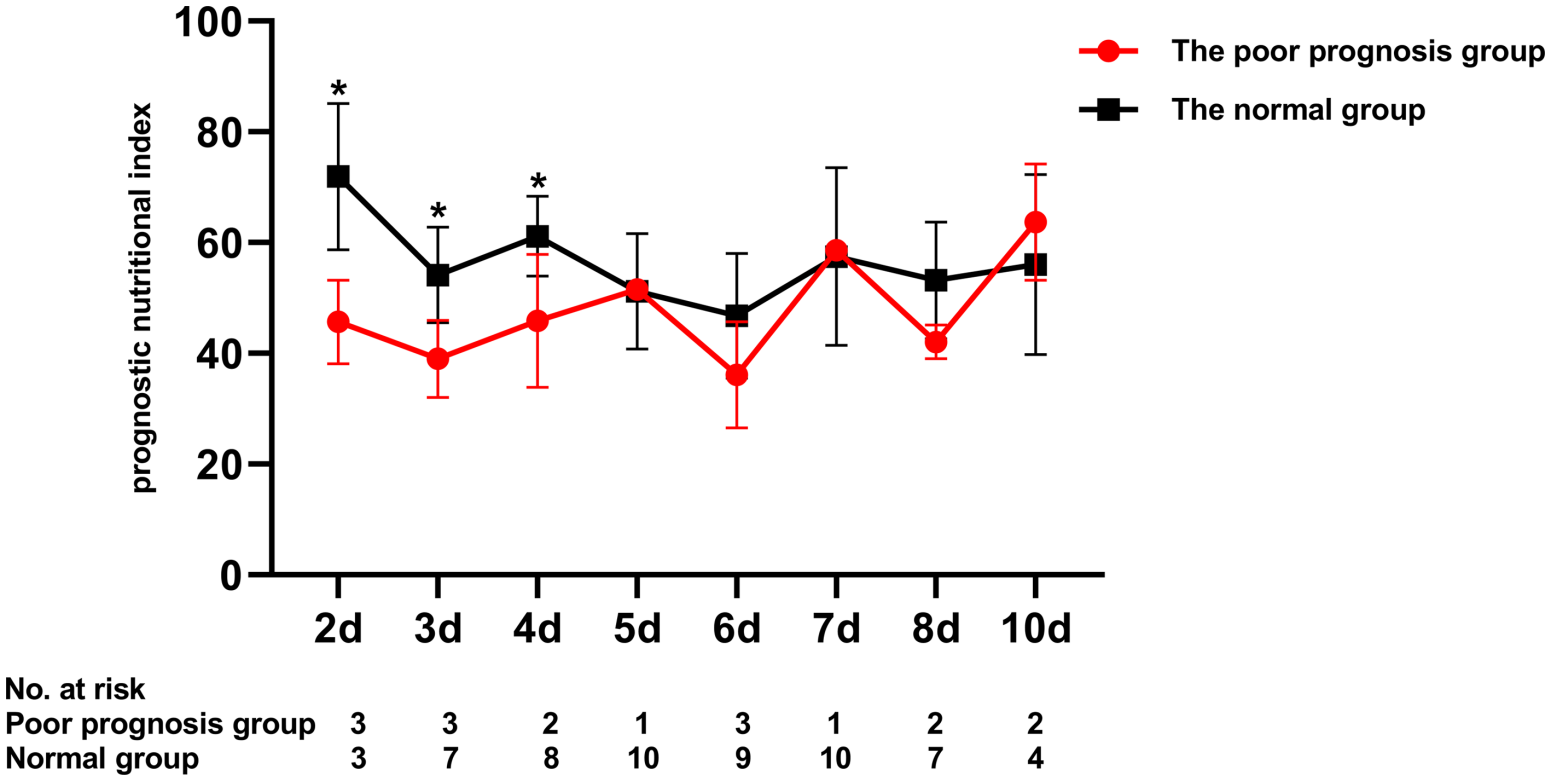
Comparison of prognostic nutritional index at different blood sampling days. *p < 0.05.

To assess the differential contributions of the two risk factors to the prediction of persistent CAA, we calculated the ROC curves for both risk factors. The 40.2 PNI cutoff value was 41% sensitive and 92% specific [area under the ROC curve (AUC), 0.708; 95% CI, 0.569–0.847; *p* = 0.009]. A 4.705 cutoff value for the maximum *Z*-score at 1 month of onset was 88% sensitive and 91% specific (AUC, 0.915; 95% CI, 0.846–0.984; *p* < 0.001; Figure 1). Kaplan–Meier survival analysis revealed that the estimated median time of aneurysm persistence was significantly higher in patients with a PNI value of ≤40 than in those with a PNI value of >40 (hazard ratio, 2.958; 95% CI, 1.601–5.464; *p =* 0.007; Figure 2). In terms of the sampling-time specific PNI for predicting persistent CAA, there were no significant differences in PNI values between patients with and without persistent CAA when sampling took place ≥5 days after fever onset. No PNI values were calculated for days 1 and 9 in the persistent CAA group because of the small sample size. The PNI was significantly different between patients with and without persistent CAA when sampled on days two (45.68 [39.15–53.90] vs. 71.92 [60.95–86.55], *p* = 0.040), three (39.00 [34.05–47.00] vs. 54.15 [41.30–65.45], *p* = 0.028), and four (45.88 [37.40–54.35] vs. 61.15 [48.15–71.00], *p* = 0.041) after disease onset (Figure 3).

## 4. Discussion

In this retrospective study, we investigated the predictive ability of the PNI values for CAA regression among patients with KD for the first time. We found that PNI may serve as a promising predictor for persistent CAA. In addition, a lower PNI indicated greater difficulty for patients to recover from coronary artery damage and more coronary arteries involved in thrombosis. Furthermore, sampling-time stratification showed that PNI obtained within 4 days from fever onset may possess greater predictive power for patients with persistent CAA.

CAA is a serious cardiac complication arising from KD and is becoming the leading cause of acquired heart disease in children (1). Consequently, the exploration of risk factors for IVIG resistance and CAA development has become a hotspot for research on KD, as patients with no response to IVIG therapy are at a high risk of CAA development. In this study, the incidence of IVIG resistance was 16.4%, and the incidences of CAD and CAA were 34 and 23.5%, respectively, similar to the findings of previous reports (31–36). However, the incidence of CAA was slightly higher than the average range in China (20.6%). Aside from the use of *Z*-scores instead of the absolute values of the coronary arteries’ internal diameter, the difference in the incidence of KD and CAA in different regions, referral bias, and epigenetic predisposition should also be considered. Our hospital is a tertiary care center; as such, it receives many referred patients with severe KD or treatment failure, including during the study period. Additionally, the genetic contribution of polymorphisms to the incidence of CAA was explored in our follow-up studies as there are many ethnic minorities in our region, and ITPKC genes’ polymorphisms were found to be associated with CAA formation in the native population (unpublished data). Furthermore, the incidence of IVIG resistance was higher in patients with CAA than that in those without CAA; however, it was not statistically significant. Notably, a significant difference was observed in the corticosteroid treatment between patients with and without CAA, indicating patients with repeated IVIG resistance may be more likely to develop CAA, consistent with previous studies (37, 38). Unexceptionally, we also found that the CAA group had a higher proportion of patients aged <1 year, a higher proportion of male patients, and a higher prevalence of complete KD, which are all risk factors for IVIG resistance and are associated with CAA development in patients with KD (39–43). Our observations suggest that continuous attention and more frequent follow-ups are required in these patients.

As body fat is associated with energy balance and inflammatory and immune responses, nutritional status plays an important role in the maintenance of health and anti-inflammatory status (44). Overweight/obesity is a pro-inflammatory state and is associated with increased cardiovascular risk (45, 46). Conversely, BMI was associated with coronary vasoreactivity, and reduced coronary vasoreactivity appears to be one of the earliest abnormalities in the development of coronary artery disease (47), whereas KD is an inflammatory-induced immune vasculitis, mainly involving the coronary arteries. In this study, statistically significant differences in nutritional status based on BMI were not observed among patients with or without CALs, regardless of injury severity. However, it is noteworthy that overnutrition is significantly associated with CAA persisting for 2 years in the univariate analysis (*p* = 0.034), but not in the multivariate analysis (*p* = 0.090), suggesting that some laboratory indicators may modify this association. Based on the literature review, this study is the first to discuss the differences in nutritional statuses of patients with CAA, with or without recovery. Nevertheless, based on the present results, we cannot draw any definitive conclusion regarding overnutrition as a risk factor for persistent CAA. As an increasing number of children with KD are reaching adulthood, there is a growing source of information regarding the long-term consequences of coronary artery damage and broader cardiovascular risk. Patients with KD may be predisposed to obesity, most likely related to lifestyle factors, particularly reduced levels of physical activity (48–50). Hence, for patients with CAA, especially those who are overweight/obese, whether lifestyle intervention can improve the prognosis warrants future investigation.

Another noteworthy finding is that a lower PNI was highly associated with CAA persisting for 2 years in addition to a higher maximum *Z*-score at 1 month of onset, which was already observed in our previous study (51). Serum albumin is known as an indicator of host inflammatory status, and hypoalbuminemia indicates a low baseline nutrition status (18, 52). However, lymphocytopenia is regarded as the marker of depressed cell-mediated immunity (53), which can lead to the development of systemic inflammatory response syndrome and is closely associated with immunity and inflammation. PNI, which takes into account serum albumin and peripheral blood lymphocytes, has been reported to be an independent predictor for malignancy or vital organ failure mortality. It can also predict the clinical outcomes of pediatric patients after cardiac surgical procedures (54–57). Moreover, PNI is known to be used as an adjunctive predictor of IVIG resistance and CAA development in patients with KD, which has been confirmed by previous studies in the last 2 years (19–23). Unfortunately, in the present study, we could not find significant differences between the subgroups based on the coronary status in terms of PNI. One reason for this discrepancy may be that different standards for groups based on *Z*-scores for coronary arteries, which might have led to different outcomes. Furthermore, the differing characteristics, age-related and genetic variations among the included patients, and time points of detection might also be responsible for the discrepant results. However, PNI was observed to be negatively associated with CAA persisting for 2 years, and further analyses suggested that patients with PNI values of ≤40 were more likely to have a longer recovery time of coronary arteries over the 2-year follow-up period. Furthermore, PNI obtained within 4 days from fever onset may possess greater predictive power for patients with persistent CAA. Although missing data and the small sample size (persistent CAA group) prevented us from investigating the role of sampling-time-specific PNI cutoff values in predicting persistent CAA, these findings provide important evidence regarding the discrepancies in the predictive values for IVIG resistance and CAA development by the same parameter. Interestingly, the lowest PNI value in both the persistent CAA and the normal groups occurred on day 6, which is consistent with the results of our previous studies and other reports (23, 58–60). Thus, pediatricians and cardiovascular specialists should focus not only on patients with CAA with a higher maximum *Z*-score at 1 month after onset, but also on patients with low PNI levels, as these can predict persistent CAA within a few days after illness onset. A previous study showed that a 10-year risk of myocardial ischemia and probability of aneurysm persistence increased among patients whose medium- and large-sized CAA regression occurred more than 2 months later (61). In the present study, the incidence of thrombosis was significantly increased in patients with CAA persisting for 2 years, and in our prior study, the same was true for patients without CAA regression within 1 year (51). The AHA guidelines defined different management scenarios for children with KD depending on the regression time of the coronary artery aneurysm (1). All the above demonstrates a delayed regression of coronary dilation, which indicates a more severe coronary vasculitis and consequently more aggressive therapy and monitoring. Dual antiplatelet therapy to prevent thrombosis is only recommended for children with medium- and large-sized CAA according to the AHA guidelines (1). However, the incidence rate of CAA stenosis or occlusion varies from 34.6 to 61% in children with KD on monotherapy with low-dose aspirin, indicating that aspirin alone may not be enough to prevent thrombosis in high-risk patients, even in those with small CAA (62–64). The medical management of patients at high risk for coronary artery thrombosis and stenoses from persistent CAA depends on the judicious use of thromboprophylaxis, and this study provides evidence regarding the potential value of risk stratification among patients with early-stage CAA, which may help identify high-risk patients requiring targeted intervention. To the best of our knowledge, this study is the first to investigate the association between PNI and CAA regression in patients with KD, suggesting that the likelihood of CAA regression might not be related simply to the original CAA size but also to nutritional condition. Future randomized controlled trials are necessary to establish different cutoff values of PNI based on sampling-times to predict persistent CAA in patients with KD.

Our study has several limitations. First, it was a single-center retrospective study, and the findings from our dataset may not be generalizable to other ethnicities. Second, the small sample size of the subgroup of patients with KD and CAA persisting for 2 years and missing data in the sampling-time limited the statistical power. Third, despite its widespread use in China, BMI has received significant criticism regarding its accuracy in defining nutritional status since it does not provide information about body fat distribution, and lipid levels were evaluated in a few patients and were not included in the statistical analysis in our study. Moreover, the regression of CAA as detected through echocardiography might occur earlier than that at follow-up, and a 2-year follow-up period may not have been sufficient to determine the outcomes of all patients with CAA. Hence, these findings should be interpreted with caution, and studies with large sample sizes and different populations are needed to confirm our findings.

## 5. Conclusion

A lower PNI was found to be an independent risk factor for CAA persisting for 2 years in patients with KD, aside from the maximum *Z*-score at 1 month of onset. Furthermore, PNI obtained within 4 days from fever onset may possess a greater predictive power for patients with persistent CAA. Another caveat that must be considered is the possible association between overnutrition and CAA regression, indicating that lifestyle interventions could be considered for improving the prognosis of patients with KD. This should also be of great future concern.

## Data availability statement

The original contributions presented in the study are included in the article/supplementary material, further inquiries can be directed to the corresponding author.

## Ethics statement

The studies involving human participants were reviewed and approved by the medical ethics committee of the First Affiliated Hospital of Guangxi Medical University (code number: 2021[KY-E-240]). Written informed consent for participation was not provided by the participants’ legal guardians/next of kin because written informed consent was waived as this was a retrospective study.

## Author contributions

JL and DS drafted the manuscript, contributed to the data collection, performed the statistical analysis, approved the final manuscript as submitted, and contributed equally to this work. PY provided the figures, contributed to the data collection and study design, and approved the final manuscript as submitted. YH contributed to the data collection and approved the final manuscript as submitted. BY administered primary treatment to these patients while they were admitted, contributed to the study design, and approved the final manuscript as submitted. KL prepared the tables, contributed to the data collection, and approved the final manuscript as submitted. YP conceived and designed the study, contributed to the data collection, and approved the final manuscript as submitted. All authors contributed to the article and approved the submitted version.

## Funding

This study received funding from the Guangxi Medical and Health Key Discipline Construction project 2019 (19), and Guangxi Clinical Research Center for Pediatric Disease (no. GUI KE AD22035219).

## Conflict of interest

The authors declare that the research was conducted in the absence of any commercial or financial relationships that could be construed as potential conflicts of interest.

## Publisher’s note

All claims expressed in this article are solely those of the authors and do not necessarily represent those of their affiliated organizations, or those of the publisher, the editors and the reviewers. Any product that may be evaluated in this article, or claim that may be made by its manufacturer, is not guaranteed or endorsed by the publisher.

## Abbreviations

A/G: Albumin-to-globulin
AHA: American Heart Association
AUC: Area under the ROC curve
aOR: Adjusted odds ratio
BMI: Body mass index
CAA: Coronary artery aneurysms
CAD: Coronary artery dilation
CI: Confidence interval
CAL: Coronary artery lesions
IVIG: Intravenous immunoglobulin
NCAL: No coronary artery lesion
NLR: Neutrophil-to-lymphocyte count ratio
PNI: Prognostic nutritional index
ROC: Receiver operating characteristic
SII: Systemic immune-inflammation index
